# Physical performance strongly predicts all-cause mortality risk in a real-world population of older diabetic patients: machine learning approach for mortality risk stratification

**DOI:** 10.3389/fendo.2024.1359482

**Published:** 2024-04-30

**Authors:** Alberto Montesanto, Vincenzo Lagani, Liana Spazzafumo, Elena Tortato, Sonia Rosati, Andrea Corsonello, Luca Soraci, Jacopo Sabbatinelli, Antonio Cherubini, Maria Conte, Miriam Capri, Maria Capalbo, Fabrizia Lattanzio, Fabiola Olivieri, Anna Rita Bonfigli

**Affiliations:** ^1^Department of Biology, Ecology and Earth Sciences, University of Calabria, Rende, Italy; ^2^Biological and Environmental Sciences and Engineering Division (BESE), King Abdullah University of Science and Technology (KAUST), Thuwal, Saudi Arabia; ^3^SDAIA-KAUST Center of Excellence in Data Science and Artificial Intelligence, Thuwal, Saudi Arabia; ^4^Institute of Chemical Biology, Ilia State University, Tbilisi, Georgia; ^5^Scientific Direction, IRCCS INRCA, Ancona, Italy; ^6^Diabetology Unit, IRCCS INRCA, Ancona, Italy; ^7^Unit of Geriatric Medicine, IRCCS INRCA, Cosenza, Italy; ^8^Department of Pharmacy, Health and Nutritional Sciences, University of Calabria, Rende, Italy; ^9^Department of Clinical and Molecular Sciences, Università Politecnica delle Marche, Ancona, Italy; ^10^Laboratory Medicine Unit, Azienda Ospedaliero Universitaria delle Marche, Ancona, Italy; ^11^Geriatria, Accettazione geriatrica e Centro di ricerca per l’invecchiamento, IRCCS INRCA, Ancona, Italy; ^12^Department of Medical and Surgical Science, University of Bologna, Bologna, Italy; ^13^General Direction, IRCCS INRCA, Ancona, Italy; ^14^Clinic of Laboratory and Precision Medicine, IRCCS INRCA, Ancona, Italy

**Keywords:** type 2 diabetes, short physical performance battery, older, mortality, machine learning, decision tree analysis

## Abstract

**Background:**

Prognostic risk stratification in older adults with type 2 diabetes (T2D) is important for guiding decisions concerning advance care planning.

**Materials and methods:**

A retrospective longitudinal study was conducted in a real-world sample of older diabetic patients afferent to the outpatient facilities of the Diabetology Unit of the IRCCS INRCA Hospital of Ancona (Italy). A total of 1,001 T2D patients aged more than 70 years were consecutively evaluated by a multidimensional geriatric assessment, including physical performance evaluated using the Short Physical Performance Battery (SPPB). The mortality was assessed during a 5-year follow-up. We used the automatic machine-learning (AutoML) JADBio platform to identify parsimonious mathematical models for risk stratification.

**Results:**

Of 977 subjects included in the T2D cohort, the mean age was 76.5 (SD: 4.5) years and 454 (46.5%) were men. The mean follow-up time was 53.3 (SD:15.8) months, and 209 (21.4%) patients died by the end of the follow-up. The JADBio AutoML final model included age, sex, SPPB, chronic kidney disease, myocardial ischemia, peripheral artery disease, neuropathy, and myocardial infarction. The bootstrap-corrected concordance index (c-index) for the final model was 0.726 (95% CI: 0.687–0.763) with SPPB ranked as the most important predictor. Based on the penalized Cox regression model, the risk of death per unit of time for a subject with an SPPB score lower than five points was 3.35 times that for a subject with a score higher than eight points (P-value <0.001).

**Conclusion:**

Assessment of physical performance needs to be implemented in clinical practice for risk stratification of T2D older patients.

## 1 Introduction

The incidence of type 2 diabetes (T2D) has reached epidemic proportions globally, and it is rapidly growing resulting in a great clinical and economic burden for the healthcare systems over the world (1, 2). T2D is managed through the implementation of complications prevention plans based on lifestyle and dietary modifications usually followed by metformin monotherapy and, when necessary, the further addition of an increasingly complex array of therapies, including oral and injectable medications (1, 2). These prevention plans are aimed at reducing the microvascular (e.g., retinopathy, neuropathy, nephropathy) and macrovascular (e.g., ischemic heart disease, peripheral vascular disease, and cerebrovascular disease) complications that exert a strong impact in terms of morbidity and mortality of T2D patients (3). In addition to microvascular and macrovascular complications, older adults with T2D are at higher risk of developing geriatric syndromes leading to important cognitive or functional impairments (4). Accordingly, while in older adults with intact cognitive and physical functions the implementation of prevention plans should pursue the same targets adopted for younger adults, in many other older diabetic patients, their implementation is complicated and strongly depends on the cognitive function, depression, physical disability, comorbidities, and degree of frailty (5–7). Therefore, several clinical parameters should be taken into account when planning therapeutic strategies for complications risk reduction in the older patients: (i) older T2D patients are at high risk of adverse drug reactions consequent to the kidney and liver function disorders; (ii) the impaired adrenergic alert symptoms makes the incidence of asymptomatic hypoglycemia more frequent in this population setting; (iii) the lower medication adherence of older patients with respect to younger people (8). For these reasons, the promotion of healthy ageing in T2D patients requires a comprehensive age‐related healthcare approach, as advocated by the latest ADA standard of care recommendations for this age group (2, 9). On the basis of these recommendations, it is a priority to establish methodologic approaches to individualize prevention plans in order to avoid overtreatment in frail patients and under treatment in other groups of patients (6), recommending different glycosylated hemoglobin targets and glucose-lowering therapies focused on patient preferences, needs, and risk profile. The ADA recommendations, however, are difficult to implement because modern T2D care systems require integrated care between general practitioners, diabetologists and geriatricians, and other members of the healthcare team. Often, older T2D patients are excluded from the personalized treatment targets.

Several studies have identified the risk factors associated with survival in T2D patients younger than 70 years (10–14). Among these, the duration of diabetes, hypertension, hypercholesterolemia, and poor glycemic control were the most relevant (10, 15, 16). However, there is no clear evidence that such factors might also influence the risk of mortality of older diabetic patients. To date, there are few comprehensive data on the risk factors related to survival at the oldest ages and their interaction with indicators of frailty in T2D patients (16–18).

The aim of the present study is to evaluate the link between chemical-clinical risk factors, geriatric parameters, and mortality in T2D patients aged ≥70 years.

## 2 Materials and methods

### 2.1 Study population

This retrospective longitudinal study was conducted in a real-world sample of older T2D patients attending outpatient facilities of the Diabetology Unit of the IRCCS INRCA Hospital of Ancona (Central Italy) between January 2014 and October 2015. The study was approved by the Ethical Committee of the IRCCS INRCA Hospital, Italy (reference number: CE-INRCA-18013), with a waiver of informed consent as retrospective deidentified data were used. As the study was retrospective, the objectives of glycemic control, the correction of risk factors, and treatments have been managed by diabetologists according to their usual clinical practices in agreement with the official Italian recommendations (Italian standards for the treatment of diabetes mellitus, *Standard Italiani per la cura del diabete mellito*, joint project of Italian Association of Diabetologists, *Associazione Medici Diabetologi*, AMD and Italian Diabetes Society, *Società Italiana di Diabetologia*, SID) (19). In accordance with the AMD-SID recommendations, to better personalize the treatment of older patients, a comprehensive geriatric assessment has also been routinely performed (7). Patients with type 1 diabetes, secondary diabetes, acute disease stage, or severe intercurrent illnesses were excluded from the present study.

### 2.2 Data collection

The following baseline data were recorded:

**-** *general characteristics*: demographic data, anthropometric data, medical history, clinical laboratory parameters;

**-** *diabetes features*: diabetes duration, glycated hemoglobin, fasting glycemia, microalbuminuria, diabetic complications, antidiabetic drugs;

**-** *geriatric assessment:*

**-** Short Physical Performance Battery (SPPB): SPPB is a standardized measure of global physical function that has been validated in frail elderly people and predicts a wide range of clinical outcomes. It has three components: a standing balance test, a walking speed test (walking 4 m), and a strength test (assessed by the time it takes to get up from a chair five times). Each component is scored from 0 to 4; the sum of the scores ranges from 0 to 12, with lower scores indicating more severe physical dysfunction (20, 21);
**-** Activities of daily living (ADL) define the level of dependence/independence in six daily personal care activities including bathing, toileting, feedings, dressings, urine, and bowel continents and transferring (in and out of bed). The summary score ranges from 0 (low function, dependent) to 6 (high function, independent) (22);
**-** Instrumental activities of daily living (IADL) assess instrumental daily activities such as telephone, shopping, food preparation, housekeeping, doing laundry, taking medicine correctly, and transfers. The summary score ranges from 0 (low function, dependent) to 8 (high function, independent) (23);
**-** Mini-Nutritional Assessment (MNA) is a tool to assess the nutritional status of elderly individuals. The collective score of MNA ranges from 0 to 30. The MNA scores ≥24 are considered normal nutrition (24);
**-** Short Portable Mental Status Questionnaire (SPMSQ) is used for the assessment of organic brain deficit in elderly patients. Scoring: 0–2 errors: normal mental functioning, 3–4 errors: mild cognitive impairment, 5–7 errors: moderate cognitive impairment, 8 or more errors: severe cognitive impairment (25);
**-** mini Geriatric Depression Scale-5-items [mini GDS-5 items] is a tool for the investigation of signs suggestive of depression. Scoring: 0–1 normal, ≥2 depression (26);
**-** EuroQuol-5 Dimensions Questionnaire (EQ-5D-5L) is a quality of life survey instrument. The EQ-5D-5L consists of two pages: the EQ-5D descriptive system (mobility, selfcare, usual activities, pain/discomfort, anxiety/depression) and the EQ visual analogue scale (EQ VAS) (27).

The primary outcome of the study was all-cause 5-year mortality. Information on vital status has been ascertained after a mean period of 53.3 ± 15.8 months from the baseline visit, after which 209 T2D patients died (20.9%) and 778 (77.7%) were still alive, whereas for 14 patients (1.4%) vital status was unknown. The T2D cohort consisted of 987 T2D outpatients. After excluding 10 individuals with missing follow-up information, we obtained a final cohort of 977 individuals.

### 2.3 Data analyses

T2D patient characteristics were summarized using means (± standard deviations [SD]) for continuous variables and counts and percentages (%) for categorical variables. SPPB score was considered as either continuous or categorized (SPPB score 0–4 or 5–8 vs. 9–12) variable (28). Independent sample t-test, chi-squared test, and ANOVA are used when appropriate.

We used the automatic machine-learning tool JADBio to identify parsimonious mathematical models able to correctly stratify the patients according to their mortality risk (29). The tool uses a multivariate analysis approach for (a) identifying the minimal set of features needed for predicting a given outcome, (b) deriving the best predictive model based on the selected features, and (c) assessing the performance of the derived model. It employs the Bootstrap Bias Corrected Cross-Validation (BBC-CV) to provide an unbiased estimation of performance that adjusts (controls) for trying multiple ML pipelines. The classification algorithms used are linear, ridge, and Lasso regression, decision trees, random forests (RF), and support vector machines (SVMs) with Gaussian and polynomial kernels. To create a parsimonious model that can be more efficiently used in daily clinical practice, we performed feature selection by using the Statistically Equivalent Signatures (SES) included in JADBIO (30). To derive the predictive models, JADBio machine learning algorithms including penalized Cox regression models, survival decision trees, and survival random forests were employed. All steps of the analysis are cross validated, in order to fairly compute the performance of all candidate models, with an additional bootstrapping step added for removing any overoptimistic bias caused by overfitting (31).

Model performance was assessed through the concordance index (c-index) (32). The c-index computes the percentage of patient pairs that are correctly ordered by the predictive algorithm according to their time to event. Censored cases are dealt with by removing the corresponding pair whenever a meaningful comparison in terms of time to event is not possible. A c-index of 1 indicates perfect ranking of their patients according to their relative risk, whereas 0.5 indicates random risk assessment and a value < 0.5 corresponds to a model performance worse than random guessing.

The importance of the factors included in the final model is estimated by assessing the decrease in performance when the predictor is dropped from the model.

The decisions tree approach (33), a multivariate technique for both data exploration and prediction, was used to develop a predictive model for the mortality risk, SPPB score categories, and geriatric parameters. The model was divided into two groups at five split layers to produce the most division into four subgroups as possible for the mortality risk.

The statistical analyses were performed by using R Statistical Software (version 4.0.5, R Software for Statistical Computing), as well as SAS software (JMP 17.2) and JADBIO software as service platforms (https://jadbio.com). P‐values were considered statistically significant if less than 0.05.

## 3 Results

### 3.1 Clinical characteristics and survival status

The T2D cohort consisted of 977 T2D outpatients aged 76.5 (SD: 4.5) years and with 46.5% of men; individuals had a mean diabetes duration of 16.4 (SD: 11.1) years and a mean HbA1c of 7.4 (SD:1.2) %. The clinical characteristics of the whole sample of diabetic patients and of subgroups of deceased and surviving patients are reported in **Table 1**. Subjects deceased at follow-up were older, predominantly men, with longer duration of diabetes, higher blood sugar levels, higher prevalence of nephropathy, chronic renal failure, neuropathy, myocardial ischemia, and myocardial infarction compared with surviving subjects. Deceased subjects also had a higher frequency of SPPB categories with lower scores with respect to survival patients.

**Table 1:** Baseline characteristics of T2D patients stratified according to mortality.

	Dead (n = 209)	Alive (n = 768)	Total (n = 977)	P-value*
Age, years	78.7 ± 4.5	75.9 ± 4.3	76.5 ± 4.5	<0.001
Sex, male	118 (56.5)	336 (43.8)	454 (46.5)	0.001
Smoke habit				0.046
No smoker	95 (45.5)	423 (55.1)	518 (53.0)	
Former smoker	93 (44.5)	278 (36.2)	371 (38.0)	
Current smoker	21 (10.0)	67 (8.7)	88 (9.0)	
Body mass index, kg/m^2^	28.6 ± 5.1	28.7 ± 4.6	28.7 ± 4.7	0.644
SPPB categories				<0.001
0–4	76 (36.4)	138 (18.0)	214 (21.9)	
5–8	86 (41.1)	255 (33.2)	341 (34.9)	
9–12	47 (22.5)	375 (48.8)	422 (43.2)	
Fasting glucose, mg/dL	152.7 ± 55.0	143.6 ± 36.7	145.6 ± 41.6	0.025
HbA1c, %	7.5 ± 1.3	7.4 ± 1.2	7.4 ± 1.2	0.326
Diabetes duration, years	17.9 ± 11.7	16.0 ± 10.8	16.4 ± 11.)	0.028
Hypertension	162 (77.9)	602 (79.6)	764 (79.2)	0.583
Hypercholesterolemia	87 (41.8)	361 (47.8)	448 (46.5)	0.129
Retinopathy	80 (38.5)	296 (39.2)	376 (39.0)	0.856
Nephropathy	81 (38.9)	136 (18.0)	217 (22.5)	<0.001
Chronic kidney disease	68 (32.7)	104 (13.8)	172 (17.8)	<0.001
Neuropathy	80 (38.5)	194 (25.7)	274 (28.4)	<0.001
Cerebral vasculopathy	25 (12.0)	84 (11.1)	109 (11.3)	0.714
Myocardial ischemia	133 (63.9)	344 (45.5)	477 (49.5)	<0.001
Myocardial infarction	60 (28.8)	119 (15.7)	179 (18.6)	<0.001
Transient ischemic attack	13 (6.3)	37 (4.9)	50 (5.2)	0.435
Peripheral artery disease	47 (22.7)	86 (11.4)	133 (13.8)	<0.001

Data were expressed as mean ± SD, or n (%). *t-test for continuous variables; chi-square test for categorical variables.

Regarding diabetic medications, approximately one-third of the patients (35.2%) were taking sulphonylureas alone or together with other medications, 31.4% were taking insulin, almost 49.5% were using biguanides, and 11.4% were taking dipeptidyl peptidase-4 (DPP-4) inhibitors. We excluded antidiabetic drugs to the 19 variables chosen as the minimum set of predictors to estimate the risk of death of patients with T2D. The main reason for this choice is that drug therapy is periodically monitored by the medical team and adjusted according to blood glucose control and the clinical requirements of the patient. Therefore, we have considered that the inclusion of drug therapy at baseline could lead to additional biases, especially considering the 5-year follow-up window. The absence of a time-dependent drug variable did not allow us to explore the potential effects of drug treatment on the observed findings. After a mean follow-up time of 53.3 (SD: 15.8) months from the baseline visit, 209 (21.4%) patients of the first cohort died (**Table 1**).

### 3.2 Machine learning approach

**Figure 1:**
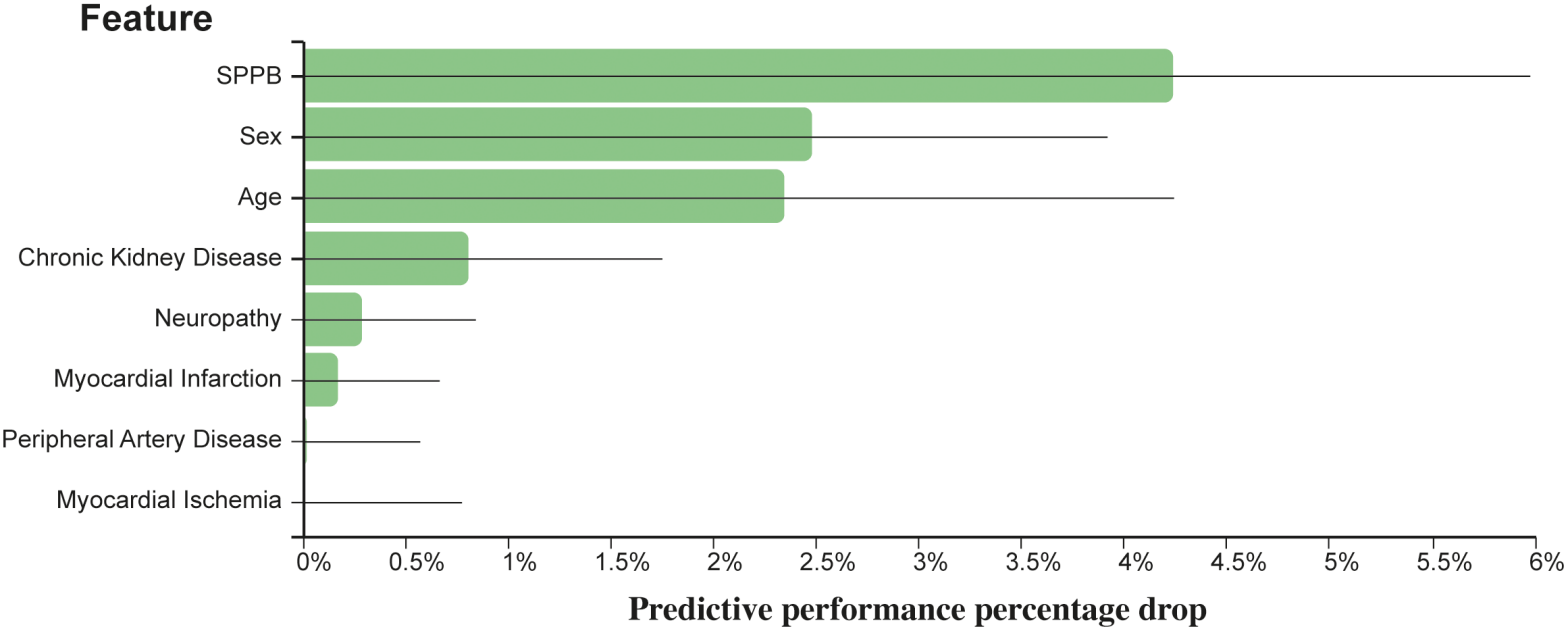
Relevance of each predictor with respect to predictive performance. The y-axis reports the reduction in concordance index that would be observed if the respective predictor is eliminated by the model.

The JADBio analysis identified eight features out of 19 variables as the minimal set of predictors needed for optimally estimating the risk of death for T2D patients. The eight features included age, sex, SPPB, CKD, myocardial ischemia, PAD, neuropathy, and myocardial infarction. A penalized Cox regression model based on these predictors achieves a 10-fold cross validated, bootstrap-corrected concordance index of 0.722 (95% confidence interval: 0.683–0.758). **Figure 1** reports the importance of each of the eight predictors in terms of their contribution to predictive performance.

The full JADBIO results are available at http://bit.ly/3ZWNFiJ. Using SPPB in its categorical form provides a model with the same features, and very similar predictive capabilities (concordance index: 0.726 [0.687–0.763]). These results are also available at http://bit.ly/3ZXJvrb.

Features are standardized before deriving the model; thus, the coefficients are directly comparable.

In summary, the SPPB score was ranked as the most important variable whose impact on mortality in terms of relative change of c-index was 4.23%. It was followed by male sex (2.47%), age (2.35%), CKD (0.81%), neuropathy (0.29%), myocardial infarction (0.17%), PAD (0.02%), and myocardial ischemia (<0.01%).

SPPB score (categorical version) was ranked as the most important variable whose impact on mortality in terms of relative change of c-index was 4.20%. It was followed by gender (2.84%), age (2.72%), CKD (0.61%), neuropathy (0.34%), PAD (0.07%), myocardial infarction (0.06%), and myocardial ischemia (<0.01%).

**Figure 2:**
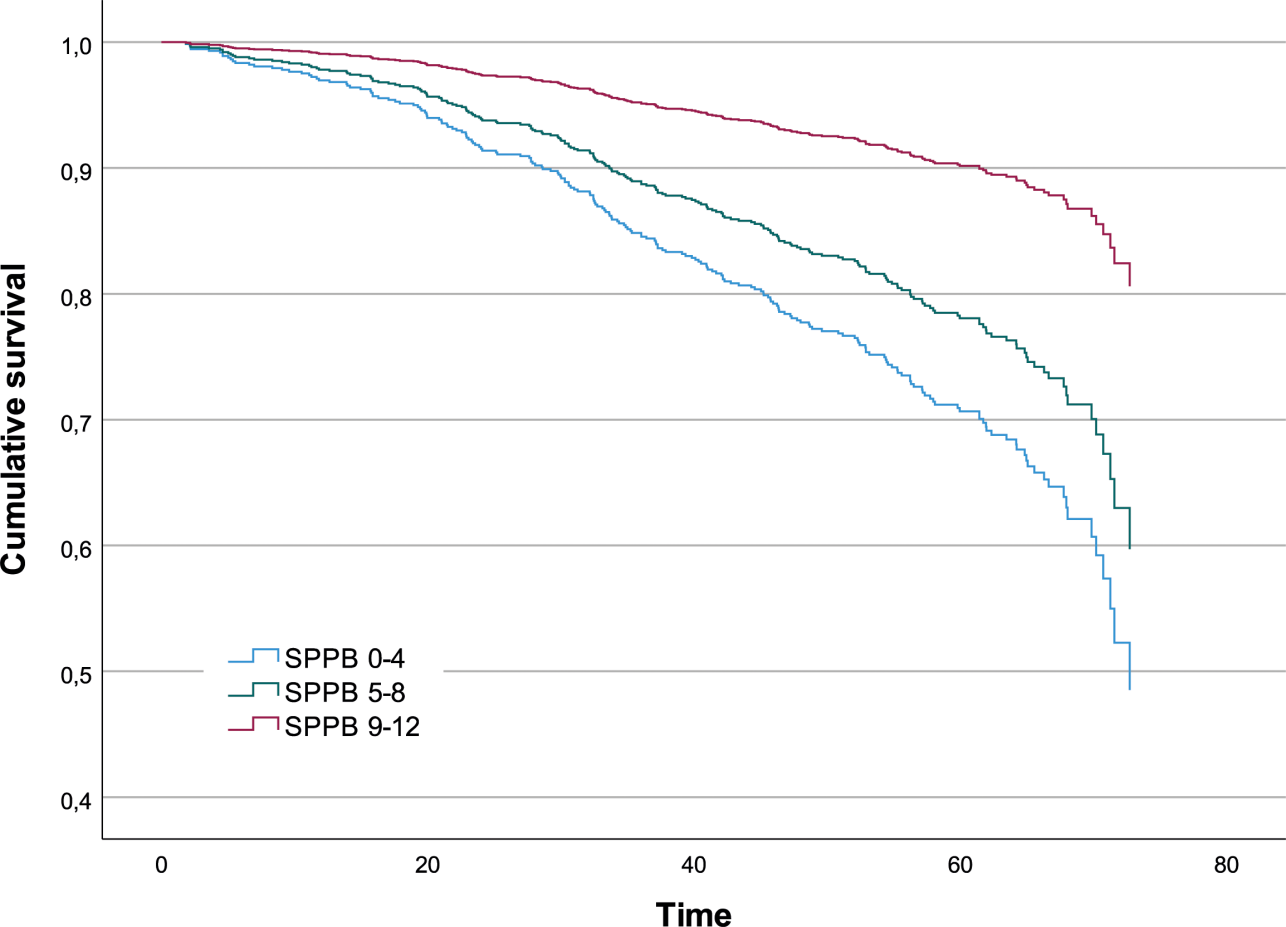
Survival function analysis by SPPB categories.

According to the Cox regression model trained on the variables selected by JADBio, the risk of death per unit of time for a subject with a SPPB score lower than five points is 3.35 times that for a subject with a score higher than eight points (P-value<0.001); the risk of death per unit of time for a subject with an intermediate SPPB score is 2.39 times that for a subject with a score higher than 8 points (P-value <0.001) (**Table 2**). In **Figure 2**, the survival functions for the three SPPB categories are reported.

**Table 2:** Cox regression model coefficients.

	coef	exp(coef)	se(coef)	z	Pr(>|z|)
SPPB 0–4	1.21	3.35	0.21	5.66	<0.001
SPPB 5–8	0.87	2.39	0.19	4.57	<0.001
Male sex	0.70	2.02	0.15	4.55	<0.001
Age	0.35	1.42	0.07	5.06	<0.001
Chronic kidney disease	0.35	1.41	0.16	2.17	0.030
Peripheral artery disease	0.31	1.36	0.17	1.80	0.071
Myocardial ischemia	0.27	1.31	0.16	1.63	0.103
Myocardial infarction	0.23	1.26	0.17	1.33	0.182
Neuropathy	0.16	1.18	0.15	1.06	0.288

SPPB, short physical performance battery.

### 3.3 Relationship between SPPB and geriatric baseline parameters

Parameters related to the multidimensional geriatric assessment across SPPB categories of T2D patients are reported in **Table 3**. T2D patients in the lowest SPPB score category were significantly older than those in the group with the highest scores and with a higher prevalence of women (P-value <0.001 in both cases).

**Table 3:** Geriatric parameters across SPPB categories of T2D patients.

	SPPB			
	0–4 (n = 214)	5–8 (n = 341)	9–12 (n = 422)	P-value
Age, years	78.9 ± 4.6	77.0 ± 4.4	74.9 ± 3.9	<0.001
Sex, male	51 (23.8)	128 (37.5)	275 (65.1)	<0.001
Diabetes duration, years	19.1 ± 12.0	17.3 ± 11.)	14.9 ± 10.5	<0.001
GDS ≥2	126 (58.1)	136 (38.1)	98 (22.2)	<0.001
ADL (at least one limitation)	48 (22.1)	86 (24.1)	116 (26.3)	0.482
IADL	4.5 ± 2.5	6.5 ± 1.8	6.7 ± 1.5	<0.001
SPMSQ ≥8	59 (27.2)	25 (7.0)	13 (2.9)	<0.001
MNA	23.9 ± 3.1	26.1 ± 2.4	27.1 ± 2.0	<0.001
Euro-Qol-5D-5L	53.9 ± 20.5	64.5 ± 17.5	71.4 ± 15.1	<0.001

Data are n (%) or mean ± SD. MNA, Mini Nutritional Assessment; ADL, activity of daily living; IADL, Instrumental Activity of Daily Living; SPMSQ, Short Portable Mental Status Questionnaire; GDS, Geriatric Depression Scale (five items); EQ-5D-5L, Euro-QoL-5D-5L test; SPPB, short physical performance battery.

T2D patients in the lowest SPPB score group had more limitations in the instrumental activity of daily living, higher prevalence of cognitive impairment and depression symptoms, and worse nutritional status and self-reported quality of life.

### 3.4 Decision tree for mortality

**Figure 3:**
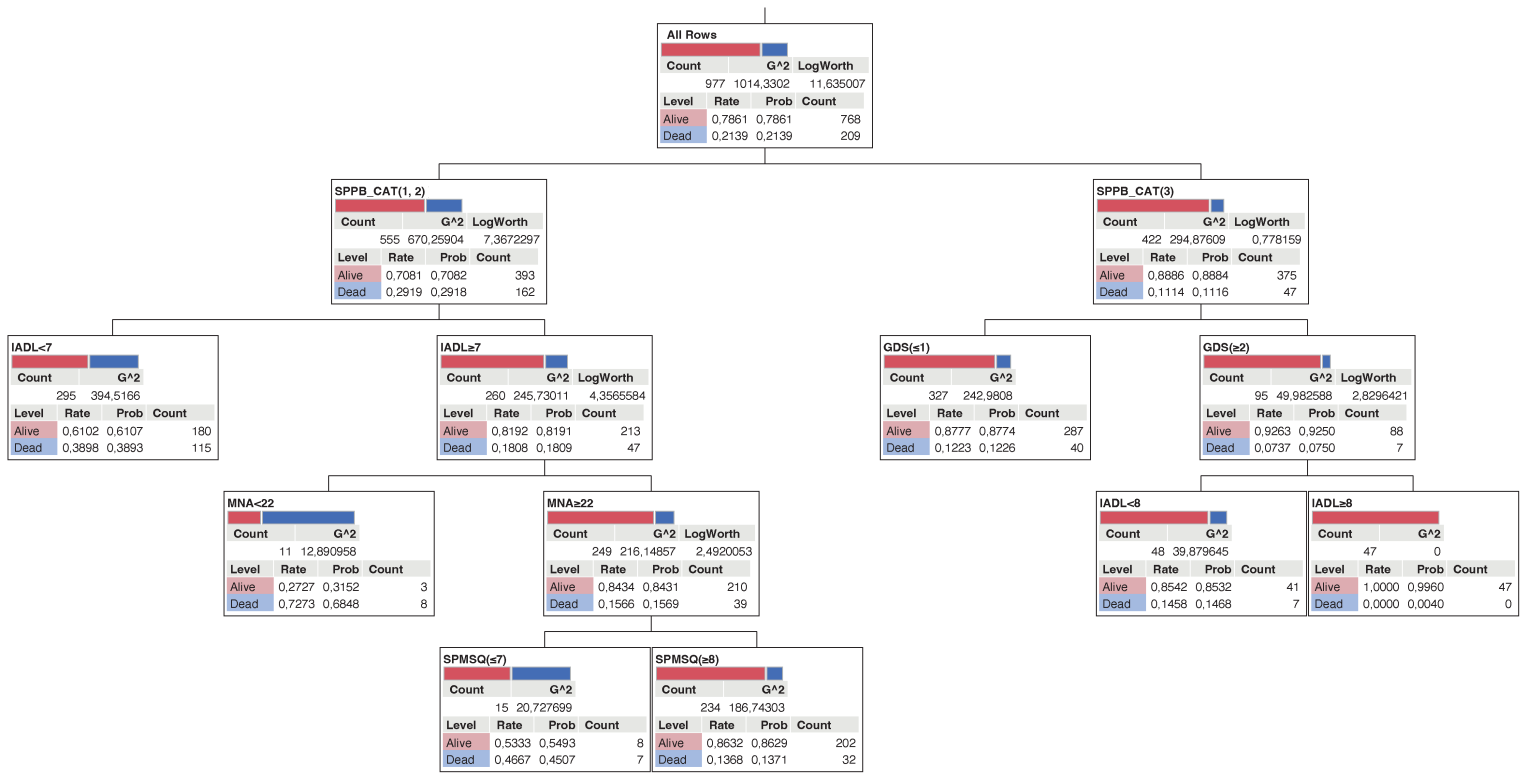
Decision tree for mortality in older diabetic subjects. The deceased and survival subjects are represented as blue and red respectively.

The decision tree with five layers identified as mortality risk factors is shown in **Figure 3**. Based on the results, in the subgroup with lower score SPPB categories (group 1 = 0–4 and group 2 = 5–8) and IADL <7, 39% of subjects were deceased (blue strip). Moreover, in the subgroup with IADL ≥7, MNA ≥22, and SPMSQ ≤7, the number of survival patients was about the same of those deceased. Finally, the subgroup with the SPPB category with the highest score (group 3 = 9–12) and GDS ≥2 and IADL = 8 included patients which are all survivors (**Figure 3**).

## 4 Discussion

T2D is a chronic disease, affecting an increasing number of older patient’s world wild. The associated micro- and macrovascular complications are drivers of morbidity and mortality. There is increasing interest in using stratification in type 2 diabetes to target resources, individualize care, and improve outcomes. The systematic reviews of studies applying population stratification in T2D identifies common themes in stratification and outcome variables, in particular a focus on HbA1c (34). The guidelines for managing diabetes in the T2D older patients recognize the need to consider frailty, comorbidities, and functionality when making decisions on patient management (35, 36). Frailty, considered as a generalized loss in physiological reserve capacity, is emerging as a high‐impact geriatric syndrome, and according to a number of studies, T2D adds a fivefold increase in the risk of frailty in middle‐aged and older people (37). Frailty is currently recognized as a complication of diabetes that may subsequently account for the unexplained disability excess seen in older diabetic populations (38).

However, in the clinical practice of diabetes care, especially in outpatient clinics, physical performance measures are not always performed routinely because of several practical issues such as limited consultation time, space, and manpower. Although the biological processes that underlie frailty are still unclear and likely to be complex and multifactorial, sarcopenia may play an important role in the accelerated decline in leg lean mass, muscle strength, and functional capacity seen in older people with diabetes compared with those without diabetes (39, 40).

The aim of the present study is to evaluate the interaction between diabetic features and parameters related to geriatric assessment, on the mortality risk prediction in a large sample of T2D outpatients aged ≥70 years. Since a prediction model derived from real-world data should be an important tool for managing the setting of older T2D patients, we applied an innovative analysis performed through the automatic machine-learning (ML) tool JADBIO, to identify the mathematical models able to correctly stratify the patients according to their mortality risk. ML has been slowly entering every aspect of our lives, and its positive impact on biomedical challenges is under investigation. JADBIO analysis identified eight features such as age, sex, SPPB, CKD, myocardial ischemia, PAD, neuropathy, and myocardial infarction as the minimal set of predictors needed for optimally estimating the risk of death for T2D patients. The SPPB is a well-established tool to assess lower-extremity physical performance status (39). In addition to the non-modifiable parameters such as age and sex, the SPPB test together with the evaluation of the complications of the disease are useful for predicting the risk of mortality and guiding the management of the older diabetic patient on an outpatient basis (41). Large literature data have confirmed that poor performance on the SPPB is associated with an increased risk of all-cause mortality, suggesting that the systematic implementation of the SPPB in clinical practice settings should be useful prognostic information (42–44).

Recently, it was demonstrated that the assessment of the SPPB scale before hospital discharge increased the ability to predict adverse events in older patients affected by acute coronary syndrome (45). We observed that the SPPB score was the best predictor of T2D mortality with respect to the other parameters. Patients with SPPB <5 experienced a 3.42-fold increased mortality risk compared with patients with SPPB >8. As previously reported, HbA1c was not associated with mortality risk (17). T2D patients in the lowest SPPB score group were significantly more disabled and had higher prevalence of cognitive impairment and depression symptoms and showed a better self-reported health and nutritional status.

Decision tree analysis highlighted the relationship among mortality, lower SPPB categories, loss of IADL, poor nutrition, and depression symptoms.

Group-based trajectories of SPPB scores identified distinct subgroups in LIFE Study participants, and using these group assignments in outcome models, a significant association with major mobility disability was observed (46). SPPB is a parameter of physical frailty, recently included as the primary outcome measure in the REHAB-HF clinical trial on older patients with heart failure (47). We did not include other parameters of sarcopenia and frailty, as we used SPPB as the index of physical frailty and sarcopenia (48). A multicomponent intervention seems to be effective in the reduction of the incidence of disability in older adults with physical frailty and sarcopenia, characterized by SPPB scores of 3–7. These data strongly suggest that physical frailty and sarcopenia may be targeted to preserve mobility in vulnerable older people (48).

In conclusion, stratification is an important tool to optimize effectiveness and efficiency in T2D management of older patients. Targeting interventions to the highest mortality risk T2D patients may allow resources to be better used and costs to be reduced.

## Data availability statement

The raw data supporting the conclusions of this article will be made available by the authors, without undue reservation.

## Ethics statement

The studies involving humans were approved by Ethical Committee of IRCCS INRCA, Ancona, Italy (reference number: CE-INRCA-18013). The studies were conducted in accordance with the local legislation and institutional requirements. The ethics committee/institutional review board waived the requirement of written informed consent for participation from the participants or the participants' legal guardians/next of kin because the study is retrospective. Considering that the data concerned routine visits for the management of diabetes in elderly subjects and that the observation period was rather long, the ethics committee approved the study protocol, waiving informed consent.

## Author contributions

AM: Formal analysis, Writing – original draft. VL: Formal analysis, Writing – original draft. LS: Formal analysis, Writing – review & editing. ET: Writing – original draft, Data curation, Investigation. SR: Writing – original draft, Data curation, Investigation. AC: Supervision, Writing – review & editing. LS: Writing – original draft, Formal analysis. JS: Supervision, Writing – review & editing. AC: Funding acquisition, Writing – review & editing. MCo: Writing – review & editing. MCapr: Writing – review & editing. MCapa: Resources, Writing – review & editing. FL: Project administration, Writing – review & editing. FO: Writing – review & editing, Funding acquisition, Methodology. ARB: Conceptualization, Methodology, Supervision, Writing – original draft, Writing – review & editing, Data curation, Investigation.
